# Classification and Use of Natural and Anthropogenic Soils by Indigenous Communities of the Upper Amazon Region of Colombia

**DOI:** 10.1007/s10745-015-9793-6

**Published:** 2015-12-19

**Authors:** C. P. Peña-Venegas, T. J. Stomph, G. Verschoor, J. A. Echeverri, P. C. Struik

**Affiliations:** Centre of Crop Systems Analysis, Wageningen University, Droevendaalsesteeg 1, 6708 PB Wageningen, The Netherlands; Instituto Amazónico de Investigaciones Científicas Sinchi, Avenida Vásquez Cobo entre Calle 15 y 16, Leticia, Amazonas Colombia; Sociology of Development and Change Group, Wageningen University, Hollandseweg 1, 6706 KN Wageningen, The Netherlands; Universidad Nacional de Colombia, Kilómetro 2 Vía Leticia-Tarapacá, Leticia, Amazonas Colombia

**Keywords:** Soil, Amazonian Dark Earths, Manioc, Indigenous communities, Colombia

## Abstract

Outsiders often oversimplify Amazon soil use by assuming that abundantly available natural soils are poorly suited to agriculture and that sporadic anthropogenic soils are agriculturally productive. Local perceptions about the potentials and limitations of soils probably differ, but information on these perceptions is scarce. We therefore examined how four indigenous communities in the Middle Caquetá River region in the Colombian Amazon classify and use natural and anthropogenic soils. The study was framed in ethnopedology: local classifications, preferences, rankings, and soil uses were recorded through interviews and field observations. These communities recognized nine soils varying in suitability for agriculture. They identified anthropogenic soils as most suitable for agriculture, but only one group used them predominantly for their swiddens. As these communities did not perceive soil nutrient status as limiting, they did not base crop-site selection on soil fertility or on the interplay between soil quality and performance of manioc genetic resources.

## Introduction

About 70 % of the Amazon basin is composed of mainly very acid, highly weathered natural soils with poor availability of the most important plant nutrients (Richter and Babbar 1991). There are, however, small patches of anthropogenic soils known as Amazonian Dark Earths (ADEs) with completely different characteristics: ADEs are usually less acid with better cation exchange capacity and base saturation than natural soils (Glaser *et al.*^2001^). ADEs also contain more nitrogen, calcium, available phosphorus (Lima *et al.*, 2002), and organic matter; the higher organic matter content results in ADEs having better moisture-holding capacity and lower rates of nutrient leaching than natural soils (Glaser and Birk 2012).

Several archaeologists alleged that the poor fertility of Amazonian soils was an environmental limitation to socio-cultural development in the region (Roosevelt 1999; Meggers 2003; McMichael *et al.*^2012^). In contrast, others posited that about 2000 years ago Amazonian societies coped with this apparent environmental limitation when ADEs emerged; these soils permitted them to increase food production and to develop complex societies (Heckenberger *et al.*^1999^; Denevan 2003; Heckenberger *et al.*^2008^).

Soil is an important resource directly related to sustainability, especially in societies that depend largely on subsistence agriculture for their food security, such as the indigenous groups in Amazonia. Understanding how indigenous groups perceive, distinguish, classify, and use soils would help us to understand from the local perspective the potentials and limitations of soils, which outsiders may have oversimplified and therefore interpreted wrongly. Local perceptions of Amazonian soils is probably different and even more complex than expected (Balée 2003; Barrera-Bassols *et al.*^2006^), but information about how indigenous people perceive natural and anthropogenic soils is limited.

Earlier reports about how indigenous people in Amazonia identify and classify soils have merely described indigenous soil classes (Wilshusen and Stone 1990; WinklerPrins and Barrera-Bassols 2004; Sánchez *et al.*^2007^) but not soil uses or people’s preferences. Most recent studies on Amazonian soils have focused on how indigenous and *Caboclo* (of Amerind-Euro or Amerind-Euro-Afro descent) people recognize and describe ADEs, failing to take into account surrounding natural soils or merely making brief references to them (German 2004; Schmidt and Heckenberger 2009; Fraser *et al.*^2012^) .

In the Colombian Amazon region, ADEs have been reported along the Caquetá River (Herrera *et al.*^1992^; Mora 2003), along some small tributaries of the Amazon River (Morcote-Ríos and Sicard 2012), and along the Putumayo River (J.A. Echeverri, unpublished data). Most of the indigenous inhabitants of the Colombian Amazon basin have access to both natural soils and ADEs. For the Middle Caquetá River region where most ADE studies have been conducted, reports show that indigenous people recognize ADEs as the soils most suitable for agriculture (Galán 2003; Andoque and Castro 2012). Studies on native production systems, however, reported that indigenous people used uplands on Oxisols and alluvial soils (floodplains), but these studies did not report the use of ADEs (Eden and Andrade 1987; Calon and Kuiper 1993). Reports on ADE uses in the Brazilian Amazon basin indicated that, wherever human settlements were located near ADEs, people used them for subsistence or market-oriented production (Hiraoka *et al.*^2003^; Fraser *et al.*2011b). There is no reason to think that indigenous people from the Middle Caquetá River region might be an exception.

The research question, therefore, is how indigenous people from the Middle Caquetá River region of Colombia classify and use natural and anthropogenic soils. To address this question, we conducted semi-structured interviews with open-ended questions, participatory observation, and field observations with four ethnic groups that inhabit the Middle Caquetá River region of Colombia. We used an ethnopedology approach (Wilshusen and Stone 1990; WinklerPrins and Barrera-Bassols 2004) to assess, understand, and interpret the way indigenous people classify and use soils on the basis of their own understanding and preferences.

## Material and Methods

### Study Area

**Fig. 1:**
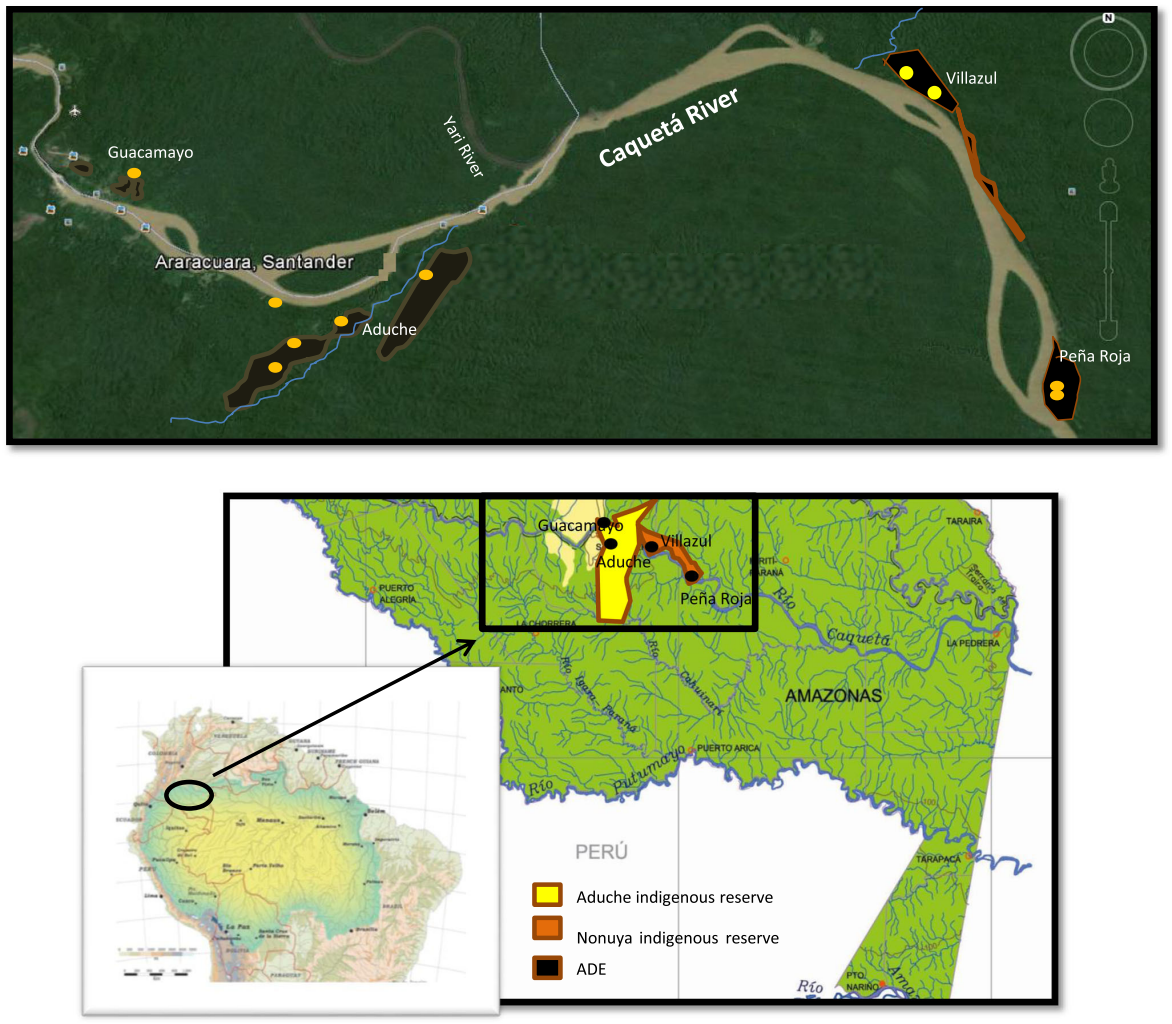
Map of the study area including the location of Aduche (in *yellow*) and Nonuya (in *orange*) Indigenous Reserves, indigenous communities, anthropogenic soils (areas in black), and communities’ *malokas* (*yellow dots*). Note: The illustration is based on the map of the Amazon basin elaborated by the Amazon Cooperation Treaty Organization (ACTO) (2008). The hydrographic map of the Colombian Amazon region was elaborated by the Instituto Amazónico de Investigaciones Científicas Sinchi (2002), and the image of the study area was taken from Google Earth (2014)

The research was conducted in the Middle Caquetá River region, on the border between the Colombian states of Amazonas and Caquetá. The area is located between 00°22′14.9″ S and 00°55′11″ S and between 72°06′36.3″ W and 71°26′18.3″ W (Fig. 1). This region is formed by the intersection of sedimentary plains of Tertiary origin (dissected terraces and hills), with rocky outcrops of Paleozoic origin running to the north creating elevated plateaus, and crossed by the alluvial planes of the Caquetá River and its tributaries. Elevation ranges between 200 and 300 m, with slopes between 7 and 25 %, and average annual rainfall is 3000 mm (Duivenvoorden and Lips 1993). December, January, and February are the driest months of the year, with 150 mm of rainfall per month on average.

On both sides of the river, there are two small semi-urban municipalities: Araracuara at the northern border of the Caquetá River with a population of 1637 (60 % indigenous people) and Puerto Santander at the southern border of the Caquetá River with a population of 2373 (67 % indigenous people) (López 2009). The two municipalities have basic infrastructure for health care, education, and commerce. In addition, Araracuara has a basic airport infrastructure where weekly flights constitute the main connection between the region and the rest of Colombia. With the exception of these two municipalities, the study area is situated in indigenous reserves.

The fieldwork was done in four indigenous communities: Aduche, Guacamayo, Peña Roja, and Villazul (Fig. 1).

The Aduche and Guacamayo communities share the Aduche Indigenous Reserve (area: 62,178 ha) located on both banks of the Caquetá River, excluding the Puerto Santander and Araracuara municipalities. The Aduche community is located mainly on transitional soils between the colluvio-alluvial valleys of the Caquetá and Aduche Rivers and the denudation surfaces. These soils are found in well-drained areas with slopes of 1 to 7 % in which colluvio-alluvial sediments have recently been deposited (IGAC 1979). The Guacamayo community, founded in 1967, is located behind Araracuara. The landscape is rich in rocky formations of sedimentary origin, some of them with petroglyphs. Soils are superficial and limited by the bedrock. The presence of quartz gravy developed soils with sandy textures and clayey soils in deeper strata (IGAC 1979). In addition, the Caquetá’s riverbanks provide the Aduche and the Guacamayo with extensive areas of floodplains.

The Peña Roja and Villazul communities share the Nonuya Indigenous Reserve (area: 59,840 ha), created by the communities after their arrival in the Middle Caquetá River region. They are located on elevated terraces of the Tertiary plateau, facing the Caquetá River. Both have access to islands and extensive areas of floodplains irrigated by the Caquetá River. The Villazul community, founded in 1956, is located about 50 km from Araracuara downriver. Peña Roja is the youngest community. It was founded in 1990 when Nonuya people living in Villazul decided to establish a Nonuya community to revive their culture (Echeverri and Landaburu 1995).

### Population

Aduche, Guacamayo, Peña Roja, and Villazul are populated by Andoke, Uitoto, Nonuya, and Muinane—ethnic groups with a common origin. They denominate themselves as *Gente de centro* (People of the Centre) in reference to their interfluvial origin between the Caquetá and Putumayo Rivers. The study area corresponds traditionally to Andoke territory and has been occupied by them since before the eighteenth century (Franco 2002), with an occasional migration forced by the rubber boom between the 1900s and 1930s. The other three ethnic groups were displaced from their original territories by the rubber boom and arrived in the Middle Caquetá River region around the 1950s.

Indigenous settlements along the Middle Caquetá River are composed of individual family houses and one or more *malokas.*^1^ The Aduche community consists of 128 persons belonging to 27 Andoke families, living in small habitational sub-nuclei around six malokas (one for each remaining Andoke clan). The Guacamayo community consists of 153 persons belonging to 34 families, living in houses distributed across Araracuara. The single existing maloka is managed by a Uitoto man, son of one of the founders of the community. The Peña Roja community consists of 71 persons belonging to 15 families living in houses distributed along the banks of the Caquetá River. They have two malokas, each one managed by one of the sons of the community founder. The Villazul community consists of 77 persons belonging to 17 Muinane families organized in a unique small habitational nucleus with two malokas managed by the sons of the founder of the community.

The populations of these ethnic groups were strongly diminished by the rubber boom in the late nineteenth and early twentieth century. Population estimates before the rubber boom were about 10,000 Andoke, 15,000 Uitoto, 1000 Nonuya, and 2000 Muinane inhabitants, according to the records taken by Thomas Whiffen during his journey in the region between 1908 and 1909 (Andrade 1986). After the rubber boom, their populations decreased to about 30 Andoke, 300 Uitoto, 4 Nonuya, and 10 Muinane inhabitants, according to estimations made by members of the communities. The Nonuya and Muinane ethnic groups, where only a few men survived, broke the tradition of marriage between clans of their own ethnic group only, and made new agreements to marry women from other ethnic groups (Orlando Paky, personal communication). This allowed them to increase the numbers in their ethnic groups and perpetuate their cultures. Although these communities became more multi-ethnic in the eyes of outside observers, they followed their own traditions and saw themselves as mono-ethnic communities.

For this research, each community was asked to suggest farmer families who knew their territory well, knew most about soils, and were active farmers. Nine families from Aduche, 10 from Guacamayo, six from Peña Roja, and eight from Villazul (33, 29, 40, and 47 % of each community’s total population, respectively) were selected for the sample, conserving the representativeness of each ethnic group.

### Permissions

This project came under the free prior informed consent agreement between the Instituto Amazónico de Investigaciones Científicas Sinchi and the communities associated with the indigenous organization Consejo Regional Indígena del Medio Amazonas (CRIMA) to work together on traditional food production as part of the process developed by the Sinchi Institute to build institutional politics with indigenous communities (Acosta and Mendoza 2006). Soil sampling was done by the Sinchi Institute under the new legislation for research institutes associated with the Ministry of Environmental Issues of Colombia (Decreto 1376 of 2013), whereby the Sinchi Institute does not need permission for genetic resources assessment when the material collected is only for research without a commercial interest.

### Fieldwork

From September 2011 to September 2013, communities were visited eight times to collect information, discuss preliminary results, and make field observations in the swiddens. These visits lasted one or two weeks, working with each community between 2 and 4 days, day and night, for a total of 90 days. Because in indigenous communities men and women have different roles and manage different but complementary information, during fieldwork members of the research team participated and assumed the corresponding gender role to access male and female information. Interviews and fieldwork were planned together with local people according to the progress of the research. Participatory observations were made during fieldwork and during daily community activities in the course of the visits.

### Natural and Anthropogenic Soils in the Study Area

As a starting point for the research, an initial discussion between the communities and the research team took place about what indigenous people understand by soil. After this discussion, the communities elaborated maps of their territories, localizing the soils they distinguished. They named soils in their native language, in Spanish (their second language), or in both. In most cases, native names corresponded to words that define soil texture and/or color, but in other cases soil names were words with no direct correspondence in the Spanish language. In such cases, linguistic interpretations or translations of names were included. Uitoto and Nonuya translations were made by Juan Alvaro Echeverri, co-author of this article who speaks Uitoto and has been working in the study area for many years. Expert academic linguists could not be found for the Muinane and Andoke languages. Therefore, native Muinane and Andoke persons fluent in both their language and Spanish helped. Orlando Paky, a health promoter in the area who was educated at the Instituto Linguístico de Verano and participated in the translation of the Holy Bible into Muinane, helped with the interpretation and translation of Muinane words. Fissi, the leader of the Andoke ethnic group who is expert in the Andoke language and culture, helped with the interpretation and translation of Andoke words.

**Fig. 2:**
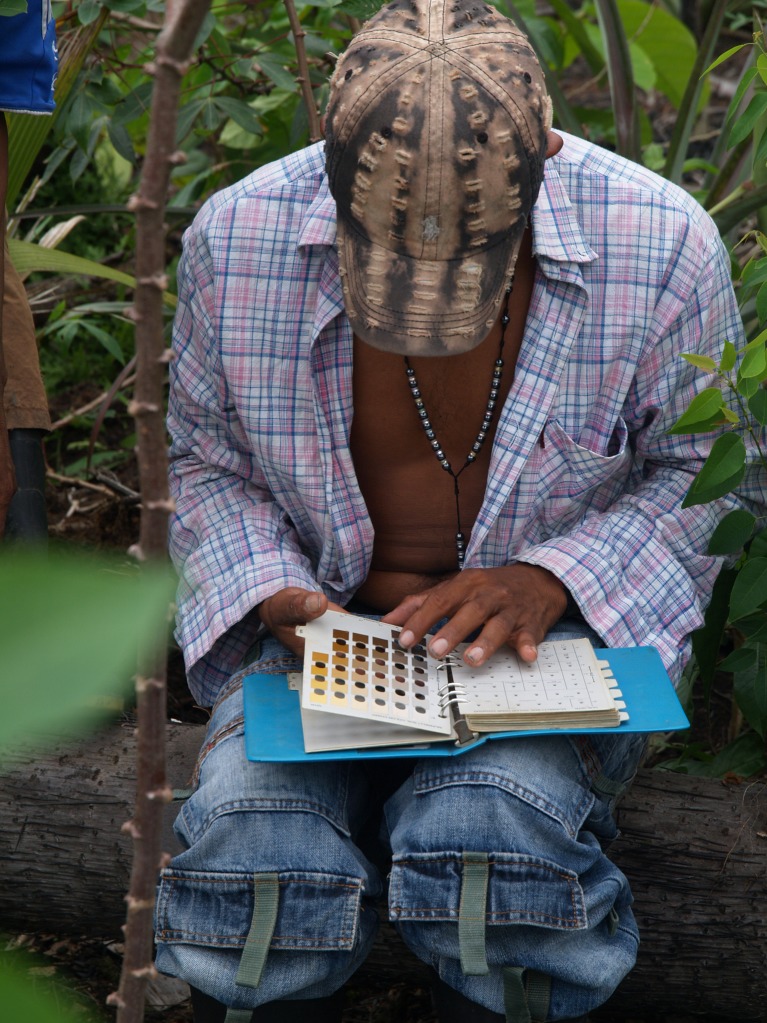
Indigenous participant assessing the color of a collected soil sample using the Munsell soil color chart. Photo: Gerard Verschoor

On the basis of the maps produced by the communities, fieldtrips with farmer families were planned to visit and describe each soil. In the field, an Edelman auger was used to collect a 90 cm deep core sample of the soil profile. GPS coordinates were taken where soil samples were collected, and a participatory description of soils was made. Soil description included the profile observation and horizon description by features observable in the field such as texture and color (using a Munsell soil color chart) (Fig. 2). Information about the soil’s recent use history, its suitability for agriculture, and crops, trees, or palms that might grow well in each soil was also collected.

About 500 g of A horizon was collected from each soil for physicochemical analyses. A total of 30 soil samples were obtained for physicochemical analyses, corresponding to a unique soil sample of a soil type or duplicate samples of the same soil type collected in different communities (Table 1). Soil samples were analyzed in the soil laboratory of the Instituto Geográfico Agustín Codazzi (IGAC) in Bogotá, Colombia. Physicochemical analyses included: texture, pH (1:1 in water), Al saturation (exchangeable Al with KCl), organic carbon (Walkley-Black), cation exchange capacity (with normal and neutral ammonium acetate), minor elements (Ca, Mg, K, Na) by DTPA, percentage of total bases, base saturation (with normal and neutral ammonium acetate), and available phosphorus (Bray II).

**Table 1:**
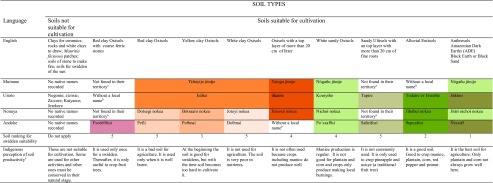
Classification of Amazonian soils by indigenous communities of the Middle Caquetá River region, soil ranking for swidden suitability and indigenous perception of soil productivity

Colors in boxes indicate the way different soils are grouped by each ethnic group

^a^The soil was not observed during the field trips for soil description in the territory of the community

^b^Indigenous people recognized and described the soil but they did not recall a native name for it

^c^Indigenous perception of each soil productivity was based on the answers given by the farmers that distinguished each one of the soils listed

**Table 2:** Percentage of settlements, agroforests, grasslands, and swiddens located on Amazonian Dark Earths (ADEs) in each of the communities visited

Use of ADE	Aduche	Guacamayo	Peña Roja	Villazul
Settling	93	6	60	100
Agroforest	100	10	100	100
Grassland	100	0	100	100
Swidden	75	0	0	27

Note: Estimates are based on observations during field trips in 2013 and reflect a relative frequency of uses and not absolute values

**Fig. 3:**
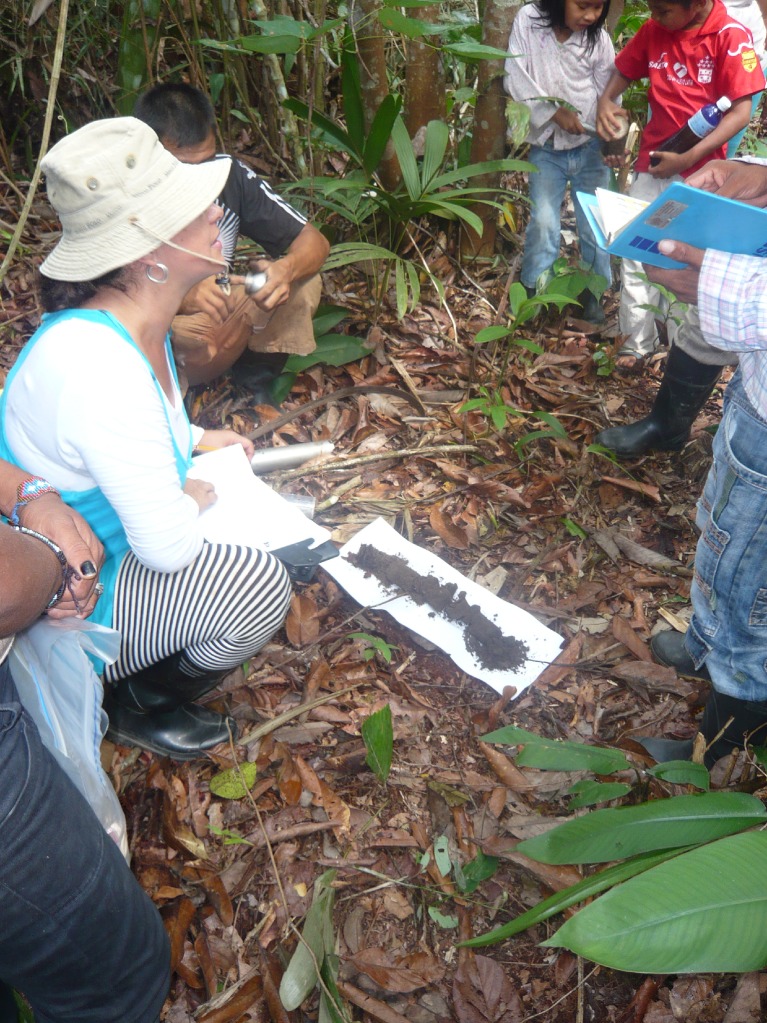
Soil description in the field with indigenous farmers of the Middle Caquetá River region. Photo: Paul Struik

After the complete soil inventory was finished in each community, each farmer ranked soils from very good (1) to very poor (5) according to his or her perception of their suitability to establish swiddens (Fig. 3). After the evaluation was finished in all communities, the 30 evaluations were grouped for a final ranking of soils. The final ranking was discussed with farmers to confirm that it represented the view of the majority.

Fieldtrips around communities were also undertaken to localize ADEs and to estimate their surface area. During fieldtrips, soils were checked superficially for color and the presence of anthropogenic materials. Information about the type of vegetation (natural or cultivated species), its age (mature forest, secondary forest, young fallow, swidden in different stages, or grassland), and the area of each ADE patch was recorded. Areas of ADE patches were estimated *in situ* with a GPS. The correct area estimation of ADE patches was difficult because they were discontinuous and had irregular shapes. To improve ADE area estimations, GPS information was compared with maps that informants people made of their territories, maps of Indigenous Reserves provided by the Instituto Colombiano de Desarrollo Rural (INCODER), and graphs of local ADEs published by Herrera *et al.* (1992) and Andrade (1983) (Fig. 1).

### Indigenous Narratives About the Origin of Soils and Their Ancient Use

Most of the information about the origin and ancient uses of soils and myths referring to these aspects were collected from traditional leaders at night in the *mambeadero.*^2^ Discussions were always accompanied by *mambe* (coca powder) and *ambil* (tobacco paste) as the essential elements for dialogue. Pre-structured trigger questions were prepared, but the research team always adopted a flexible approach about the order in which questions were asked or the order in which topics were addressed to let the traditional leaders feel comfortable answering. Because of that, not all the mambeadero sessions provided relevant information for the research, and more mambeadero sessions were required than originally expected to obtain the reported information.

### Swidden Location, Estimates of Soil Productivity, and Indigenous Perceptions of Soil Productivity

On the soil maps elaborated in each community, farmers indicated the number of swiddens they had, where they were located and on which type of soil. Information for 2011 and 2013 was recorded.

Preliminary research was undertaken on the swidden production system in the study area to understand its particularities. The information collected helped to identify variables used to estimate soil productivity. Three variables were thus identified in the three main groups of soils as ranked by indigenous farmers: fallow duration, swidden area, and time between planting and harvesting manioc (*Manihot esculenta* Crantz). These variables were evaluated in 20 swiddens (five in Peña Roja, eight in Guacamayo, five in Aduche, and two in Villazul), of which nine were located on Oxisols and Ultisols, four on Alluvial Entisols, and seven on ADEs (Anthrosols).

A fallow period starts when swiddens are abandoned after a cropping period because labor effort is no longer compensated by production due to the increased presence of weeds and the depletion of soil nutrients. The fallow duration reflects the extent of nutrient removal from the soil during the cropping period and the time required to restore nutrient stocks to a minimum to allow production of a new crop. In soils with limited nutrients, it is expected that long periods are needed to restore them without human intervention. In more fertile soils on the other hand, nutrient depletion is less and fallows can be shorter. Fallow duration was estimated in years based on local farmers’ knowledge about when the place was used before, for how long, and how many years the soil was rested before a new swidden was established. If people indicated that the patch was a primary forest never logged before, the fallow period was assumed to be at least 100 years.

A relationship between swidden size and soil fertility is expected. Larger fields are needed on less fertile soils with lower productivity than more fertile soils. The swidden area (in square meters) was estimated in the field with a GPS.

Better plant nutrition is reflected in faster plant growth. Manioc, the main crop planted in swiddens, is a good indicator to evaluate the relation between plant growth and soil productivity. In manioc, better plant nutrition results in more active nutrient translocation to roots and therefore in early root bulking (Alves 2002). When root bulking is early, farmers can harvest manioc early, thus shortening the crop cycle. A short cycle can be advantageous for the preparation of certain products produced in large quantities. The length of the cycle between manioc planting and harvesting was estimated for 20 permanently monitored swiddens because indigenous criteria determined the time between planting and harvesting. Because swidden harvesting on floodplains is influenced by floods, swiddens located on *restingas* (Alluvial Entisols on high floodplains that are reached only by high floods) were monitored in the hope that farmers would make the manioc harvesting decision on the basis of manioc root bulking rather than on the flooding regime. Indigenous farmers cultivated around 12 different manioc landraces per swidden indiscriminately on Oxisols, Alluvial Entisols, or ADEs. Consequently, the time of manioc harvesting was determined in the context of a specific manioc landrace or of multiple manioc landraces that farmers considered ready for harvesting.

Fieldtrips to swiddens were also used to ascertain indigenous perceptions about swidden productivity. These perceptions included both expectations and problems in relation to swidden production.

### Manioc Inventories

During fieldtrips to swiddens, inventories were made of the manioc landraces managed by each community. Portfolios of communities’ landraces were compared in relation with the type of soil on which the swiddens were located. A manioc landrace was defined as a unique combination of morphological characteristics clearly recognized by local people and identified by a local name. Landraces were classified by the indigenous groups into three main groups: sweet maniocs (those with non-toxic roots that can be consumed after being cooked without a previous detoxification treatment), white bitter maniocs also known as maniocs “to grate” (toxic landraces, white to very pale yellowish-colored roots, used to obtain starch after being grated), and yellow bitter maniocs (toxic maniocs with yellow-colored roots whose complete biomass is used in different preparations). In addition to fieldtrips, during research-team visits to communities, researchers shared meals or were involved in their preparation, and these were important opportunities to observe culinary traditions.

### Statistical Analysis

A one-way ANOVA Kruskal-Wallis test for non-parametric data was applied to the 20 registers obtained for fallow duration, swidden area, and number of months between manioc planting and harvesting in the three groups of soils ranked by indigenous farmers. A Chi-square test was undertaken to assess differences in manioc inventories among ethnic groups. Differences were considered significant at *p* ≤ 0.05. All statistical analyses were performed with the analytical software Statistix 9.0.

## Results and Discussion

### Indigenous Classification of Soils

The indigenous people of the Middle Caquetá River region have a complex view of the world. They understand living and non-living elements as being composed of physical and spiritual components. All elements, including humans themselves, are equally important parts of a unique unit, the world. Because all components are equally important, they cannot be isolated from one another. In this way, soil does not exist *per se*. It is part of a “place” that includes also other elements such as vegetation, water sources, landscape, and animals that live there in an integral way.

Although different elements exist and interact in the world, each element has particular characteristics that confer on the place attributes to be used for specific purposes. Indigenous people classify soils into two main groups: soils suitable for cultivation and soils not suitable for cultivation (Table 1). Soils not suitable for cultivation commonly manifest cultural (taboos), physical, or chemical constraints for agriculture. Nevertheless, they have important roles in the maintenance of environmental equilibrium. Examples of these soils according to Uitoto people are the *Kaiyanɨe* or “soils of stone to make fire;” the *Jetekore* or swiddens of the sun in which wild animals find fruits to eat; and the *Zaɨkore*, which are soils permanently swamped and covered by broad patches of *Mauritia flexuosa* palm whose fruits are an important source of food (mainly proteins and oil) for wild animals.

The indigenous people of the Middle Caquetá River region recognize two distinct layers (horizons) of soil suitable for cultivation: one formed by the litter layer and the first layer of dark earth (A horizon for soil scientists), which they denominate as *workable soil*. The second layer, formed by the deeper soil and the bedrock (B horizon, deeper mineral horizons, and bedrock for soil scientists), they denominate as *dead soil*. The indigenous discrimination of workable soil and dead soil corresponds well with the function of each layer in relation to plant nutrition, resource capture, and agricultural practices as perceived by natural sciences. The workable soil is the one in which indigenous people produce food, and it is also the fertile portion of soils. For soil scientists, it is the layer where organic matter transformations occur, and it is susceptible to degradation or improvement by human agency.

In soils with high turnover rates of organic matter, such as most of the natural Amazonian soils generally, the topsoil plays an important role in plant nutrition (Serna-Chavez *et al.*^2013^). Microbial activity is found to be restricted mainly to the first 20 cm of these soils (Peña-Venegas *et al.*^2007^). The term dead soil, on the other hand, describes well the almost non-existent biological activity in soil layers below the A horizon, where very old and leached materials coming from a predominantly kaolinite bedrock with low natural cation exchange capacity provide hardly any nutrients to plants.

**Fig. 4:**
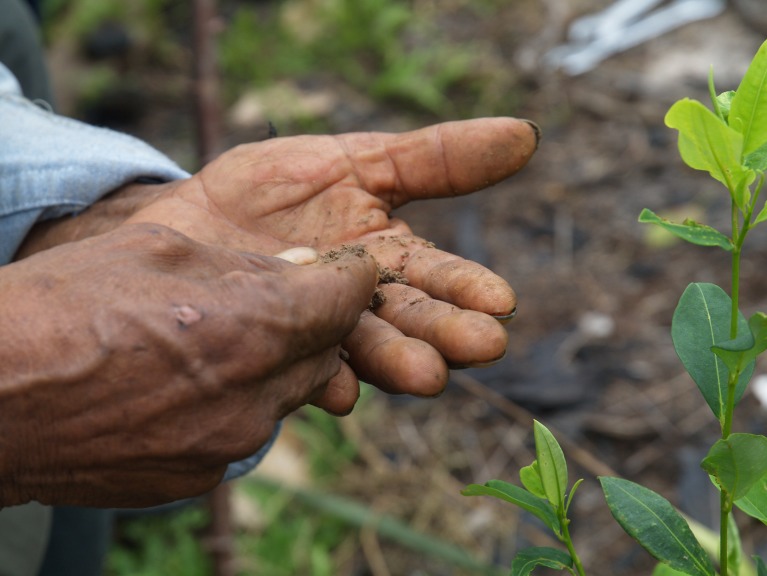
An indigenous farmer evaluating soil texture with his hands to classify the soil. Photo: Gerard Verschoor

In the research area, informants recognized nine different soils suitable for cultivation, although the quality of some of them restricts their use to sporadic occasions (Table 1). Indigenous soil classifications are based on soil texture, soil color, and the presence of other easily observable features in the field (Fig. 4). Each ethnic group classifies soils differently. Muinane people have the simplest soil classification based mainly on texture; they recognized two main groups of soils: clayey and sandy. The Uitoto also classify clayey soils in one group, but recognize differences among sandy soils, grouping them separately and using specific names for each. The Nonuya base their soil classification on both texture and color. Nonuya native names refer to texture and color characteristics followed by the word *nokea*, which means soil (*tierra* as they translate it into Spanish). Nonuya people distinguish differences between clayey soils but group all soils with a sandy texture into one group. The Andoke particularly recognize each soil individually. The Andoke language does not use a specific word for “soil” and names each soil using a specific word.

All indigenous groups recognize ADEs. They call it *Tierra negra* or *Arena negra* in Spanish (black earth or black sand in English), but they also have native names for it. For the Nonuya and Muinane ADE was one of the soils classified into the group of sandy soils, using a common word to denominate sandy soils in general (*Nógañu jiinɨje* in Muinane) or adding a word to highlight the dark color of ADEs (as in Nonuya in which the word black, *jitɨrɨ*, is added to the words *nichoɨ nokea* that mean sandy soil).

Native soil names provide information beyond merely texture or color. The Uitoto word *ɨɨkanɨe* used to denominate a soil with a thick layer of litter also means fish odor according to the *nɨpode* dictionary developed by Griffiths et al. (Griffiths, T., Coleman, J., Morales, M. (2001). An Nɨpode Uitoto – Spanish – English Lexicon. Phonetics Laboratory, University of Oxford [Unpublished manuscript]) and might be related to the production of volatile substances during organic matter decomposition. This soil is easily recognized by the amount of litter. The *Tapire* (or *Zafire*) soils, as the Uitoto denominate sandy soils with a thick upper layer of fine roots, indicate the particular short and thin forest that grows on these soils. The words used by Uitoto and Andoke people to denominate ADEs have more interesting meanings. The Uitoto word *Jiɨkɨno* can be analyzed as *jiɨ-* “small” and *-kɨ* meaning both “fire” and “generation”, and *–no* (*nɨe*) is the suffix for “place (soil),” so the name could be translated as “soil with small particles due to fire or use by former generations.” The Andoke call ADEs *Ñesxaθ*, which means textually black soils, but the term indicates that the black color originates from burning. The indigenous words encapsulate information of two major elements relating to ADE formation: fire and human activity.

### Indigenous Understanding of the Origin and Ancient Use of Soils

Soil scientists agree that natural soils are formed by erosion of bedrock, the action of weather, and the decomposition of organic matter. On the other hand, scientists accept that ADEs are anthropogenic soils created by inhabitants of the Amazon region between 2000 and 500 years ago (Neves *et al.*^2004^), easily distinguished from natural soils by their chemical properties and other features observable to the naked eye such as their dark color and their deep A horizon with the presence in most cases of potsherds, lithics, and charcoal pieces left by ancient anthropogenic activities (Kämpf *et al.*^2003^). Therefore, ADEs are classified as Anthrosols or as anthropogenic soils (a term used by both natural and social sciences researchers).

The studied communities in the Middle Caquetá River region agree that natural soils were formed naturally but have a different perception of ADEs’ origin. In local indigenous understanding, ADEs have a mythical origin in which fire is an important element. The mythical Andoke tale for *Ñesxaθ* formation describes a sky festival organized by Pepái, the son of the Andoke god Nenefí, to which only the good persons living on Earth at that time were invited. During the festivities, people threw away bones of the cooked animals that opened springs of hot water that burnt the Earth. The bones of eaten animals and the bodies of bad people on Earth altered the soil and gave *Ñesxaθ* their characteristics. The Uitoto version of ADE creation also describes a time in which the world was burnt. The evidence of that episode according to the Uitoto are the black patches “that have small parts of animals or animal forms we don’t know today.”

Regarding more recent history, our informants indicated that ADEs, formed in mythical times, provided good agricultural soils. Aurelio, the traditional leader of the Guacamayo community, said that “*Jiɨkɨno* were the favorite soils of ancient people. So, when ancient people found those black patches of soil, they settled there.” Fissi, leader of the Andoke people, indicated that: “in ancient times people pulled the wood up from the soil [removed the litter and decaying wood lying on the soil] to check the soil color. When they found *Ñesxaθ*, they settled their communities there.” The practice of locating indigenous settlements on ADEs is still maintained. The Muinane, Nonuya, and Uitoto, who did not traditionally live in the Middle Caquetá River region, established their communities on ADEs.

The Middle Caquetá River region was traditionally inhabited by the Andoke clans Cacambra (a local bird species) and Cucarrón (beetle in English), which no longer exist. The current indigenous population, however, know who lived there and how much those soils were appreciated for food production. Muinane indicated that Carijonas (an almost extinct ethnic group from the Colombian Amazon region) were the oldest inhabitants of the area, whereas the Nonuya indicated that both Cacambras and Carijonas were the oldest inhabitants there. Aurelio, leader of the Uitoto, stated: “ancient people selected ADE because production was good and it could be used after short fallows without problem” [without compromising crop production]*.* Fissi pointed out “Andokes used to fight against Carijona people for those patches of dark soil. Carijona people looked for those places because they knew they were good for manioc cropping. During Andokes’ and Carijonas’ fights for *Ñesxaθ*, people were killed, their goods were destroyed, their malokas were burnt, and the winner took the territory. Andokes also fought against Carijonas to recuperate their territories and so on for many generations. The broken ceramics and artifacts are found where ancient malokas and houses existed, and they are the remains of those wars” (interview, May 18, 2012).

Scholars explain the presence of ceramics and human artifacts in ADEs as waste deposits of ancient settlements (Schmidt *et al.*^2014^). Andoke people believe those artifacts are not only burnt household waste but also remains of malokas and houses intentionally destroyed. Consequently, places where ADEs formed were not only exposed to frequent small burnings but also to periodic large burnings when complete communities were destroyed. Destruction of communities could lead to large amounts of organic matter in the soil that with burnings might produce larger amounts of charcoal and ashes than burnt household waste. Large amounts of charcoal and ashes might change the environmental conditions of soils for ADE formation or the initial beginnings of the ADE formation process, but this requires further research.

In summary, indigenous mythology indicates soil creation before human existence. Indigenous people recognize a relationship between ancient people and ADEs in which human activities and fire were important elements associated with ADEs. Caboclos from the Middle Madeira River region have a similar understanding of ADEs (Fraser *et al.*2011a). Indigenous groups currently living in the Middle Caquetá River region do not recognize themselves as the creators of ADE. Their historical memory expressed through their narratives goes to a time in which ADEs already existed, the region was densely inhabited, ADEs had a key role in food production, they were not able to recreate it, and they needed to fight for its use. The picture recalls pre-Columbian times when the region was densely inhabited (Dull *et al.*^2010^) and ADEs were used intensively.

### Today’s Use of Natural and Anthropogenic Soils in the Study Area

Most natural soils in the study area are covered with primary and secondary forest that indigenous people use for different purposes:

#### Fruit Collection

Not all species that indigenous people consume are cropped. An important number of edible plants were never domesticated or their domestication was truncated at some point in human history (Clement 1999), and people go regularly to forested areas to collect them.

#### Extraction of materials for construction

All materials used for house or boat construction, for making artifacts, furniture, and tools for food processing are obtained mainly from the forest, except metal artifacts and modern tools for hunting and fishing.

#### Collection of Medicinal Plants

As is the case for some edible species, some natural medicines are wild plants. Medicines include complete wild plants or parts of them such as roots, leaves, bark, or resins.

Most medicinal plants are neither cultivated nor maintained in anthropic environments, and people depend on forested areas to obtain them.

#### Areas for Hunting

Most hunting occurs in secondary forests where there are palms and fruit trees still producing. Additionally, specific animals are hunted in specific places such as *salados* (soils with a high concentration of salts that wild animals visit periodically) where most of the big mammals are hunted, or patches of the *Mauritia flexuosa* palm where big rodents are hunted.

#### Swidden Establishment

**Fig. 5:**
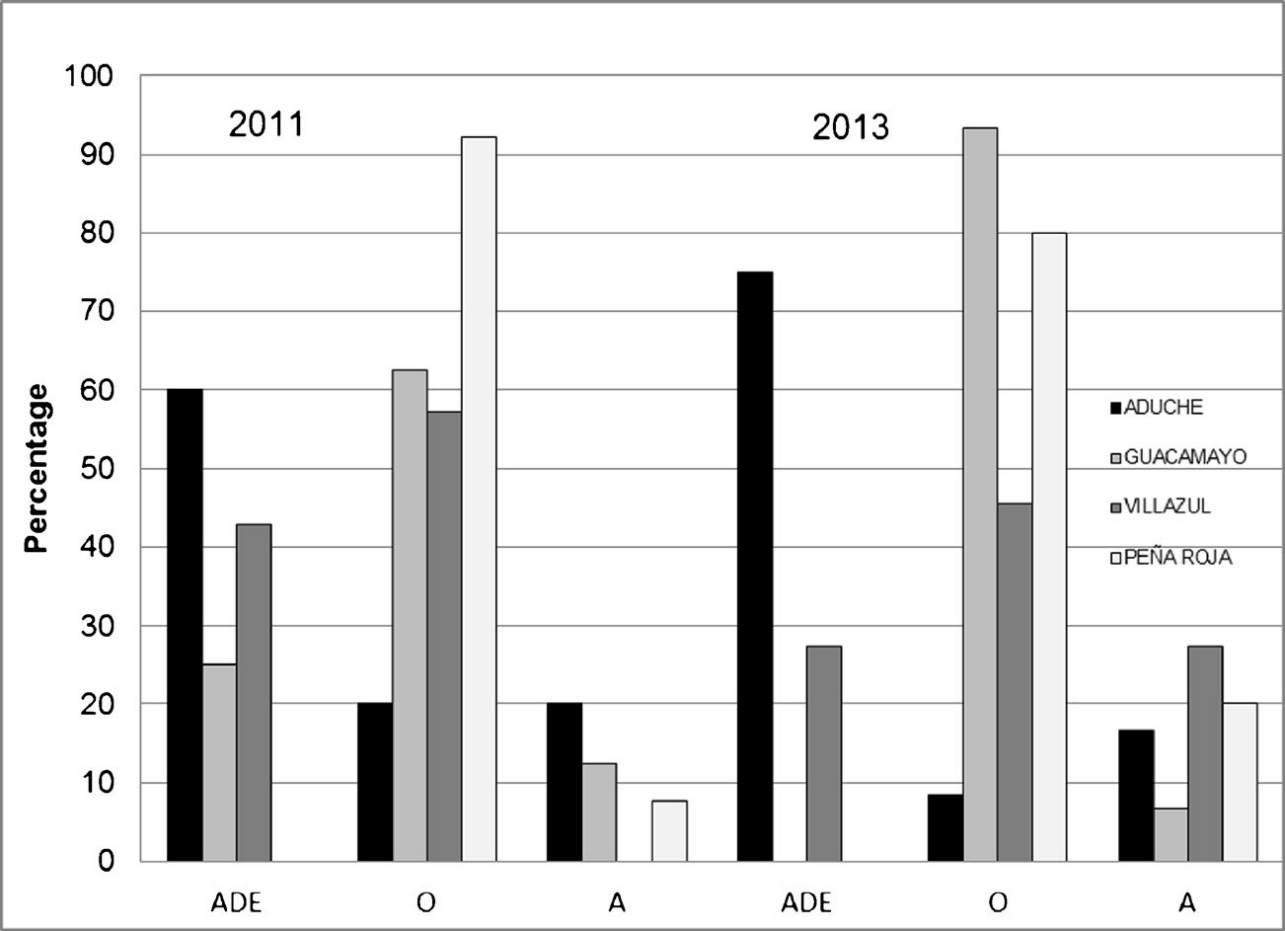
Percentage of farmers’ swiddens located on Amazonian Dark Earths (ADE), Oxisols (O), or Alluvial Entisols (A) during 2011 and 2013 in the Andoke community of Aduche, the Uitoto community of Guacamayo, the Muinane community of Villazul, and the Nonuya community of Peña Roja, Middle Caquetá River of Colombia

Between 50 and 90 % of the indigenous swiddens are located on natural soils (Fig. 5).

Common uses of ADE patches observed in the indigenous communities included:

#### Settlement

Most malokas and houses of the Aduche, Peña Roja, and Villazul communities are located on ADEs (Table 2). Indigenous people appreciate ADEs as they are good soils often with natural streams of fresh potable water in the proximity.

#### Agroforestry Systems

Swiddens observed in all types of soils were mainly of transitory crops, and almost no palms or fruit trees were planted there. Most palms and fruit trees were planted around malokas and houses on ADE, producing agroforestry systems. Agroforests included not only native trees and palms, but also exotic plants such as citrus trees, coconut palms, and mangos. A farmer from Villazul reported that “we plant fruit trees and palms in a swidden if we want to abandon and transform it into a fruit tree garden. After that, we will not log the fruit trees anymore. We will not use that place for a swidden anymore. Those places are for hunting and for fruit collecting” (interview November 27, 2012).

#### Grasslands

In the Aduche, Villazul, and Peña Roja communities, some ADE patches or parts of them near or in the settlements are covered by non-native grasses (Table 1) such as star grass (*Cynodon nlemfuensis* Vanderyst) and humidicola grass (*Brachiaria humidicola* (Rendle) Schweick). None of the communities raise cows or other domestic animals that could feed on those grasses. Periodically and especially when grasses became dry, people burn them to stimulate their re-growth. Interviews with local people indicated that men plant the patches as they like grassed landscapes. Women, however, indicated that they would prefer to use ADE patches for crop production.

#### Swiddens

Soil ranking for swiddens indicated that, by consensus, indigenous farmers consider ADEs as the best soils for food production (in the case of the Muinane, sandy soils including ADEs are ranked in first place as very good soils) (Table 1). Common expressions people use to refer to ADEs include “This soil is always preferred; it is so good; it has a lot of nutrients; the best soil; very good soil for manioc and fruits; it produces good manioc.” The soils ranked in second place were Alluvial Entisols. Other soils listed are considered as soils with limitations for agriculture but suitable for cultivation.

Most Amazonian parental materials from which soils originate are rich in kaolinite, which has limited nutrient holding capacity (Ma and Eggleton 1999). Soils’ organic matter plays an important role in cation exchange capacity (Glaser and Birk 2012). In environments with high temperatures and high humidity as is common in the Amazon region, organic matter decomposition is rapid. Nutrients liberated after organic matter decomposition are rapidly leached due to frequent strong rains and the low nutrient-holding capacity of soils, resulting in limited fertility of most natural upland soils.

According to the standard soil quality indexes for Colombian soils used by the IGAC (IGAC 1979) and the soil study done in the Middle Caquetá region by Duivenvoorden and Lips (1993), soils in the research area do not present physical problems for agriculture as all of them are loamy to sandy soils. The main constraint is their high acidity, which causes high levels of exchangeable aluminum toxic to plants, low Ca availability, and low base saturation, resulting in reduced fertility. Results of this research indicate that upland soils have a pH of 4 and an exchangeable acid saturation of over 70 %, independent of whether they are natural or anthropogenic soils. Alluvial Entisols in contrast have an exchangeable acid saturation of around 40 % (Table 3), explained by the sediment enrichment experienced every year when the Caquetá River floods (Piedade *et al.*^2001^) and deposits sediments of different mineral composition from the Andes.

**Table 3:** Physicochemical composition of nine Amazonian soils recognized by indigenous communities from the Middle Caquetá River region of Colombia

Soil name	No. soil samples	Texture	pH^h^	E.A.^i^	E.A.S.^j^	% O.C.^k^	C.E.C.^l^	Ca^m^	Mg^n^	K^o^	Na^p^	T.B.^q^	% B.S.^r^	P^s^
Red clay Oxisols with coarse ferric stones	1	LCS^a^	4	4	78	2	14	0.4	0.3	0.3	0.1	1	7	ND^t^
Red clay Oxisols	2	LC^b^ to LS^c^	3.9 (0.2)	5.0 (1.4)	89.60 (1.3)	2.0 (0.7)	13.0 (4.3)	0.2 (0.0)	0.1 (0.0)	0.2 (0.1)	0.1 (0.0)	0.6 (0.1)	4.5 (0.1)	ND
Yellow clay Oxisols	5	S^d^ to SL^e^	4.0 (0.1)	5.5 (1.5)	77.3 (16.1)	3.5 (1.4)	18.5 (3.8)	0.7 (0.7)	0.0(0.3)	0.3 (0.1)	0.0 (0.0)	1.5 (0.1)	8.3 (5.0)	18.5 (25.1)
White clay Oxisols	1	LC	4.4	5.4	89	2.1	12.1	0.1	0.1	0.2	0.1	0.7	5.5	15.7
Oxisols with a top layer of more than 20 cm of litter	3	LCS to SL	3.8 (0.5)	4.7 (4.0)	70.0 (37.5)	4.0 (2.7)	20.0 (14.1)	1.2 (1.9)	1.2 (2.0)	0.4 (0.3)	0.1 (0.1)	2.9 (4.2)	11.1 (11.7)	2.0 (3.3)
White sandy Oxisols	6	LS to SL	3.6 (0.2)	3.8 (1.3)	82.8 (7.6)	2.3 (1.3)	12.6 (7.4)	0.2 (0.1)	0.3 (0.3)	0.2 (0.1)	0.0 (0.0)	0.8 (0.4)	6.8 (3.0)	6.0 (7.4)
Sandy Ultisols with a top layer with more than 20cm of fine roots	1	LS	3.3	7.5	93.6	10.6	39.2	0.13	0.14	0.22	0.02	0.51	1.3	ND
Alluvial Entisols	5	CL^f^ to L^g^	4.4 (0.3)	3.0 (2.0)	42.1 (30.6)	2.5 (1.0)	16.4 (4.2)	3.3 (3.0)	1.4 (1.0)	0.3 (0.1)	0.1 (0.1)	5.1 (3.7)	32.8 (24.2)	4.6 (7.8)
ADEs (Anthrosols)	5	SL to LS	3.8 (0.1)	2.5 (1.3)	60.5 (22.2)	1.7 (0.6)	9.3 (4.2)	1.1 (0.9)	0.3 (0.2)	0.2 (0.1)	0.1 (0.0)	1.7 (1.1)	17.2 (7.7)	88.6 (157.2)

Texture is presented as a range and the other variables are expressed as the average and its standard deviation (SD) in parenthesis (for soils with more than one sample) according to the number of soil samples collected from each soil

Texture: ^a^Loamy clayey sandy soils; ^b^ Loamy clayey soils; ^c^ Loamy sand soils; ^d^ Sandy soils; ^e^ Sandy loam soils; ^f^ Clayey loam soils; ^g^ Loamy soils

^h^ pH (1:1 in water); ^i^ Exchangeable acidity with KCl; ^j^ Exchangeable acid saturation with KCl; ^k^ Percentage of organic carbon (Walkley – Black); ^l^ Cation exchange capacity using normal and neutral ammonium acetate; ^m^ Calcium by DTPA; ^n^ Magnesium by DTPA; ^o^ Potassium by DTPA; ^p^ Sodium by DTPA; ^q^ Total bases; ^r^ Percentage of base saturation using normal and neutral ammonium acetate; ^s^ Available phosphorus by Bray 2 in parts per million

^t^ Phosphorous availability not detected by Bray 2

Rather than differences in pH, sampled soils differ in their chemical composition. Soils with more organic carbon have higher cation exchange capacities. Soils providing better conditions for agriculture are generally those with higher Mg and K availability, a higher total base saturation, and a higher percentage of base saturation (Table 3). When ADEs and Alluvial Entisols were compared, alluvial soils had larger Ca amounts and a better chemical composition for agriculture than ADEs, but indigenous farmers rank Alluvial Entisols in second place. Periodic floods that limit the number of crops that can be produced on alluvial soils are the main factor in indigenous soil ranking. Corn and plantain, which are usually cropped in Alluvial Entisols, are complementary to the staple food manioc, and farmers do not need to crop them permanently or in large amounts to satisfy their requirements. Hence, advantages for these crops are generally not major determinants in indigenous appreciation of soils.

The chemical composition and soil fertility measures reported in Table 2 are not part of the studied communities’ environment conceptualization. They use soil color, soil texture, and vegetation as indicators of potential productivity. They understand the importance of organic matter in soil nutrient supply. They know from experience that a dark soil holds more nutrients. They also recognize which trees and which type and age of vegetation provide more and better organic matter that confers a deeper and better ‘workable soil’ for crop production. Old vegetation, with a deep ‘workable soil*’* that produced well before, is the main criterion in selecting a place for a new swidden.

Every year, each household opens a new swidden on uplands. Swiddens are cropped mainly with manioc, which occupies about 70 % of the total cropped area, other non-staple crops such as plantain (*Musa paradisiaca* L.), pepper (*Capsicum annuum* L.), pineapple (*Ananas comosus* Merr.), and corn (*Zea mays* L.); ritual species such as coca (*Erythroxylum coca* Lam.) and tobacco (*Nicotiana tabacum* L.), and some medicinal and cosmetic plants. These latter crops have short production cycles, whereas the manioc harvest starts a year after planting and continues for another 2 years. This means that each family had three to four swiddens on uplands in growing or harvesting stages, and 18 % of the families additionally had one or two swiddens on floodplains.

Because mainly ADEs and Alluvial Entisols had ‘workable soil*’* with good conditions for food production, their frequent selection for swiddens was expected. Swidden inventories made in 2011 and 2013 indicated that Uitoto, Nonuya, and Muinane farmers used predominantly Oxisols, whereas Andoke farmers used predominantly ADEs for their swiddens in the same period (Fig. 5).

People’s perceptions of ADEs likely vary depending on their history and their degree of interaction with ADEs (Fraser *et al.*2011a). ADEs are found relatively frequently in the Central Amazon. It could be assumed that, because of the relative abundance of ADEs in their surroundings, Caboclos in the Central Amazon have been more exposed and have more access to ADEs, explaining why ADEs are used more in the Brazilian Amazon than in other regions. Limited access to ADEs was therefore explored as a reason for the results described above.

### Access to ADE use

It is known that indigenous farmers will not open swiddens further than 5 km from their community because of the limited human physical capacity to transport harvested produce. All ADE patches registered were less than 5 km from the communities, and therefore ADEs were available for swiddens. Total ADE area in the Aduche reserve was estimated to be 115 ha (86 ha in Andoke territory and 29 ha in Uitoto territory), and in the Nonuya reserve to be 70 ha (distributed in almost equal areas of 35 ha for the Nonuya and Muinane). Land tenure in indigenous reserves is collective, and all community members have equal access to land. So, when ADE hectares were equally divided over the families of each group, each Andoke family had access to 3.18 ha; each Uitoto family to 0.85 ha; each Nonuya family to 2.33 ha; and each Muinane family to 2.05 ha. ADE access for Uitoto families could be less because some ADEs were located in the Araracuara municipality and used by families that were not part of the Guacamayo community. The results therefore indicated that all ethnic groups had access to ADEs, although some had access to more ADE land than others (Andoke farmers for example). However, ADE access did not explain the results, as ADE use did not correspond to access to it.

### Estimation of Soil Productivity and its Indigenous Perception

**Fig. 6:**
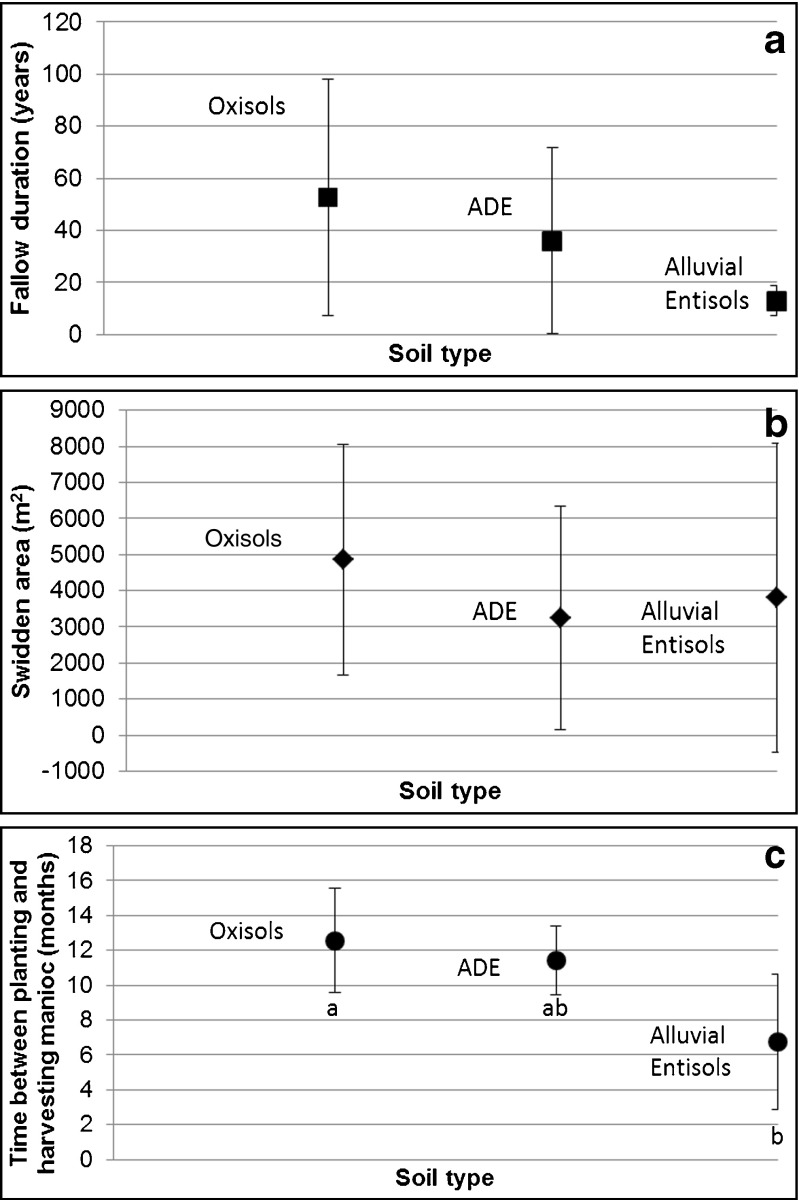
Average and standard error of fallow duration, swidden area, and time between planting and harvesting manioc in swiddens on Oxisols, Amazonian Dark Earths, and Alluvial Entisols. **a**. Fallow duration (in years) of places where swiddens were established; **b**. Swidden area in square meters; **c**. Time between planting and harvesting manioc in months (*letters under each soil* correspond to test results of pair-wise comparison)

There was a high variability in fallow duration (Fig. 6a), swidden area (Fig. 6b), and time between planting and harvesting manioc (Fig. 6c), independent of the soil in which swiddens were located. A Kruskal-Wallis one-way ANOVA test showed no significant differences in the duration of fallows (*p* = 0.3394) or in swidden areas (*p* = 0.4055) on different soils. Differences in the time between planting and harvesting manioc among soils were significant (*p* = 0.0322). Manioc grown on Alluvial Entisols was harvested earlier than manioc grown on uplands, but there were no significant differences between Oxisols and ADEs, despite differences in their chemical composition.

Indigenous farmers estimated swidden productivity based on manioc production. From 33 interviewed farmers, 64 % were satisfied with their swidden production independent of the soil selected. Only four farmers declared that they were dissatisfied with swidden production. Two farmers with swiddens on ADEs indicated that swiddens were not burnt properly and nutrients did not liberate properly into the soil, and that this affected swidden production. Two other farmers with swiddens on non-ADEs indicated that strong rains on soils with poor drainage capacity and groups of capybara (*Hydrochoerus hydrochaeris*) had spoiled a good portion of the cultivated manioc.

Studies from the Brazilian Amazon on how local farmers used ADEs indicated that Caboclos and indigenous communities chose predominantly ADEs for agriculture. Some communities face limitations in accessing ADEs, such as the Kuikuro who have to walk about 10 km to cultivate ADEs (Schmidt and Heckenberger 2009), but nevertheless continue cultivating it. In the Middle Caquetá River region, some indigenous farmers have restrictions on cultivating ADEs, but this does not explain why farmers without restrictions on ADE use are inclined to cultivate Oxisols. It is remarkable that instead of using ADEs for food production, some people prefer to keep ADEs covered with grasses for aesthetic reasons. Indigenous soil selection for swiddens therefore is not chiefly based on soil fertility, but other conditions drive selection.

### Cultural Values Associated with Soil Selection

**Fig. 7:**
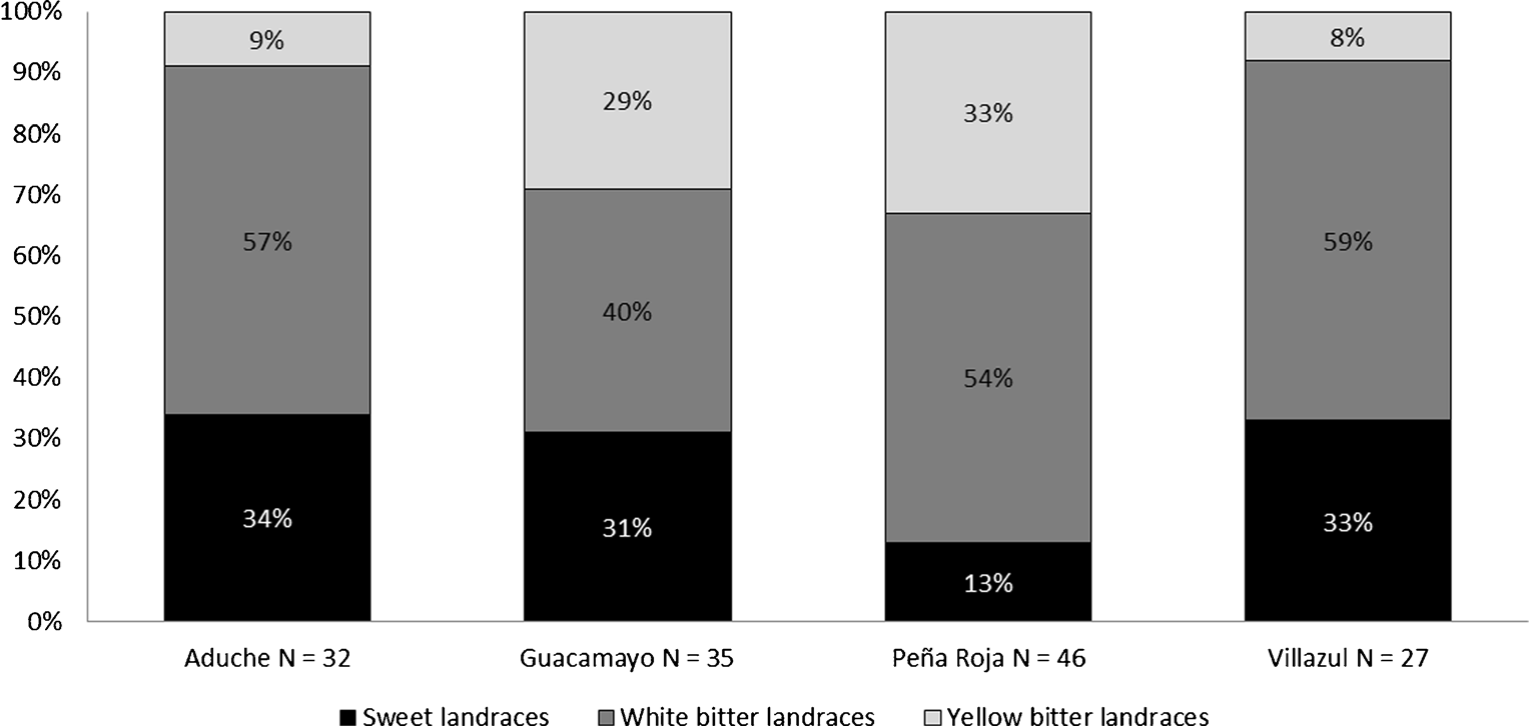
Manioc inventories (N) and percentages of sweet, white bitter, and yellow bitter landraces cultivated in the Andoke community of Aduche, the Uitoto community of Guacamayo, the Nonuya community of Peña Roja, and the Muinane community of Villazul, Middle Caquetá River of Colombia

The studied communities maintain very diverse manioc inventories of sweet, white, and yellow bitter manioc landraces (Fig. 7). When the proportion of sweet, white, and yellow bitter maniocs was compared among communities, significant differences were found in the number of sweet and yellow bitter maniocs (10.5 and 25.8, respectively, compared to a Chi-square table value of 7.82). The Andoke and Muinane cultivate the lowest numbers of yellow bitter maniocs, and they also use ADEs predominately for swiddens. The Nonuya, on the other hand, cultivate the highest number of yellow bitter maniocs and did not use ADEs for their swiddens, in either 2011 or 2013.

Differences in manioc inventories among ethnic groups were related to their culinary preferences. Nonuya and Uitoto traditionally consume *casabe de masa* (round flatbreads made with the whole fermented yellow bitter manioc root biomass). As bitter maniocs grow well on Oxisols (Eden and Andrade 1987; Wilson and Dufour 2006). Nonuya farmers do not require ADEs for swiddens and prefer to use ADEs to crop agroforests with exotic species they appreciate and that cannot be grown on non-ADEs. On the other hand, Andoke and Muinane traditionally consume manioc mainly as *casabe de almidón* (round flatbreads made from starch obtained from white bitter manioc roots). Burgos and Ceróz (2012) found that manioc cultivated in sandy soils with high phosphorus availability accumulate more starch.

In the natural soils of the Middle Caquetá River region, most phosphorus is present as Al-P or Fe-P with a low availability (Souza *et al.*^2009^). The higher phosphorus availability in ADEs and long periods of manioc growth (ADEs do not experience periodic floods as floodplains do) could allow a larger accumulation of root starch in white bitter maniocs that the Andoke and Muinane particularly appreciate, encouraging them to use ADEs more frequently for their swiddens.

## Conclusions

Soil classification by indigenous groups in the Middle Caquetá River region reflected their knowledge about local soils and their perceptions about their potential and limits for cultivation. The studied communities recognize ADEs as the best soil and agree that ADEs provide good conditions for most crops, palms, and exotic and native fruit trees, but their high appreciation of ADE production does not lead to more frequent use. In contrast, other contemporary Amazonian farmers use ADEs for food production more often than other soils (Fraser *et al.*2011b), both for subsistence but also to supply local markets. Under those conditions, soils such as ADEs play an important role in food production. Contemporary uses of natural and anthropogenic soils by indigenous communities in the Middle Caquetá River region contrast with their historical narratives, in which ADEs had a predominant role in food production. Drastic declines of these ethnic groups’ population could have changed the way ADEs are perceived. Today, the Andoke, Uitoto, Nonuya, and Muinane remain as small ethnic groups living in a region with low population densities where abundant forested areas exist with conditions good enough to guarantee their food security based on manioc, and thus are exceptional cases of how ADEs are perceived and used.

Of all Amazonian soils, ADEs are the only ones with clear legislation regarding their use because of their anthropogenic origin, whereby they are classified also as archaeological sites (both in Brazil and in Colombia). Although most ADEs have been reported in the Brazilian Amazon, they have been reported in several other countries of the Amazon basin as well. The frequent use of ADEs for agriculture in Brazil by groups traditionally occupying these soils limits to some extent their potential as archaeological sites, as this agricultural use may have largely disturbed the sites. Archaeological studies of ADEs in Colombia and Amazonian countries other than Brazil are scarce. However, these countries at the fringe of the Amazon basin may in fact contain more undisturbed ADEs and more indigenous communities who have preserved their traditions. Such sites and communities with similar perceptions of Amazon soils as those presented in this article might constitute an important opportunity to obtain information about Amazonia’s history and better understand the origin of anthropogenic soils.

## Footnotes

1 Communal indigenous round houses made of wood and with a roof of palm leaves where traditional leaders live and teach their culture to other community members.

2 The space in the *maloka* for knowledge exchange.
